# Distinguishing dystrophic calcification from calciphylaxis

**DOI:** 10.1016/j.jdcr.2023.07.015

**Published:** 2023-07-26

**Authors:** Elizabeth Jones, Alexander Valiga, Miriam Solowey

**Affiliations:** a Department of Dermatology and Cutaneous Biology, Thomas Jefferson University Hospital, Philadelphia, Pennsylvania; b Sidney Kimmel Medical College, Thomas Jefferson University, Philadelphia, Pennsylvania

**Keywords:** dystrophic calcification, nonuremic calciphylaxis, sodium thiosulfate

## Introduction

Calcinosis cutis, a disorder in which calcium salts deposit in skin and subcutis, is categorized into five subtypes: dystrophic calcification, metastatic calcification, idiopathic calcification, iatrogenic calcification, and calciphylaxis.

Dystrophic calcification, the most common subtype, typically results from local tissue damage^1^^,^^2^ and is proposed to be caused by the release of phosphate binding proteins by necrotic cells in response to tissue damage, inflammation, or hypoxia.^2^ The condition often presents with nontender nodules of the skin or subcutis and normal serum calcium.

Calciphylaxis is believed to be caused by impaired inhibition of calcification in the microvasculature.^3^ A deficiency in carboxylated matrix Gla protein, a vitamin K dependent inhibitor of vascular calcification, has been associated with calciphylaxis.^3^ Conditions resulting in vitamin K deficiency, including Warfarin usage and end stage renal disease, have been implicated.^3^ Several causes of nonuremic calciphylaxis have been reported, notably in association with alcoholic cirrhosis.^1^^,^^4^^,^^5^ Additional risk factors include hypercalcemia, hyperphosphatemia, and hyperparathyroidism.^3^

Intramural vascular calcification of small to medium-sized vessels, typically of the dermis and subcutaneous fat, is a key pathologic diagnostic criterion of calciphylaxis.^1^ Following medial calcification, subintimal fibroplasia and thrombosis result in vascular occlusion, ischemia, inflammation, and necrosis of surrounding tissue.^3^ Consequently, calciphylaxis is associated with severely painful lesions.^3^ Diagnosis requires clinicopathologic correlation as diagnostic histopathological findings of intramural calcification of small vessels and vasculopathy can be subtle.

The following case underscores how variations in the clinicopathologic presentation of dystrophic calcification and calciphylaxis can create diagnostic challenges.

## Case

**Fig 1:**
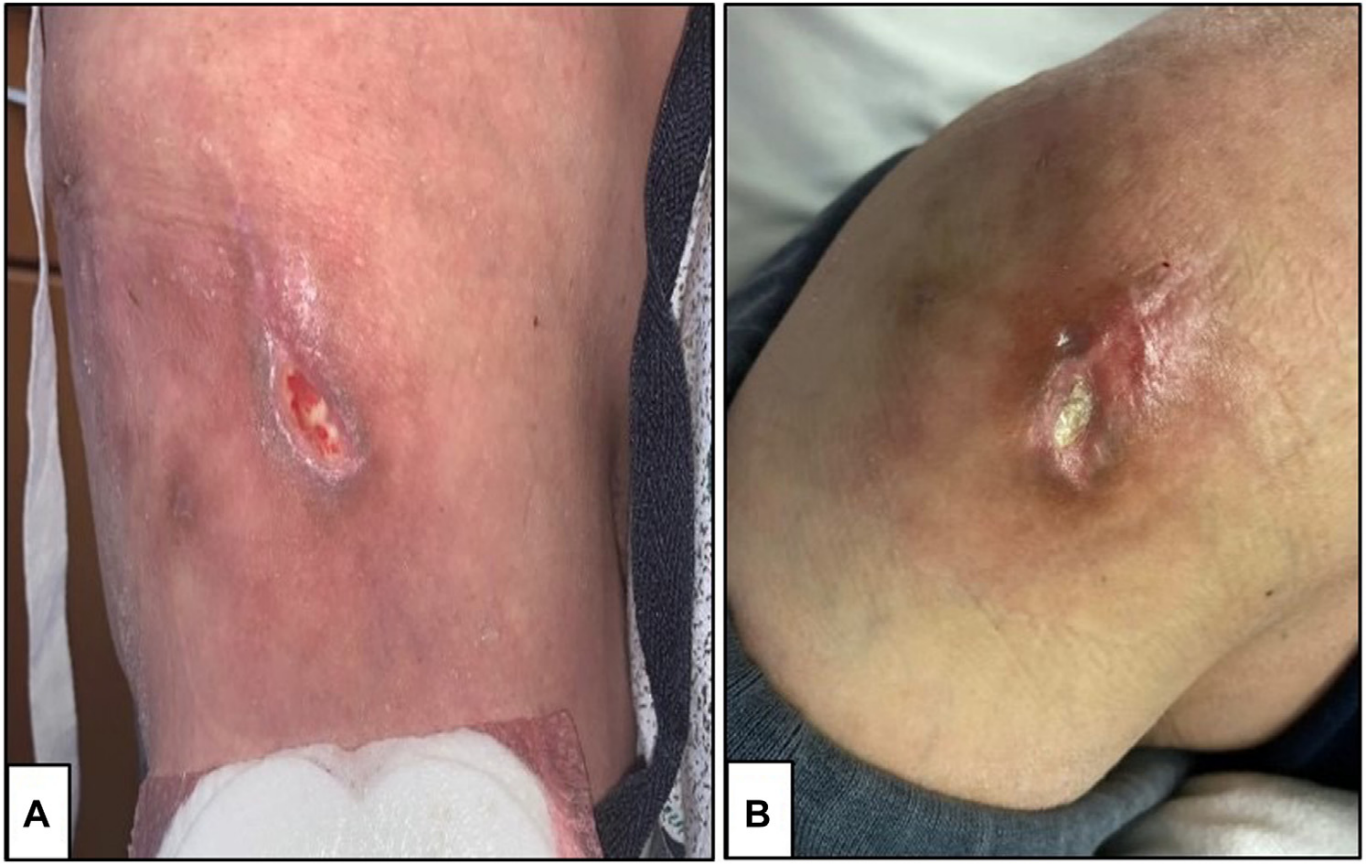
**A,** Patient’s *right* lateral hip demonstrating a single, well-demarcated ulcer with fibrinous material at its base. **B,** Partially re-epithelialized ulcer following 14 days of treatment with 3 times weekly sodium thiosulfate.

A 37-years-old woman admitted for decompensated alcoholic cirrhosis with Wernicke’s encephalopathy was found to have an intermittently tender and firm chronic ulcer on her right hip (Fig 1, *A*). The lesion, present for a year as a subcutaneous nodule, began to ulcerate. The patient had no history of recent trauma to the area. The patient’s family reported a 11.3 kg weight loss over the course of 2-3 months. An outpatient magnetic resonance imaging of the right hip 3 months prior to presentation showed a lesion with diffuse calcification of soft tissue overlying the gluteus maximus in the lateral hip. The patient was transferred to our institution.

**Fig 2:**
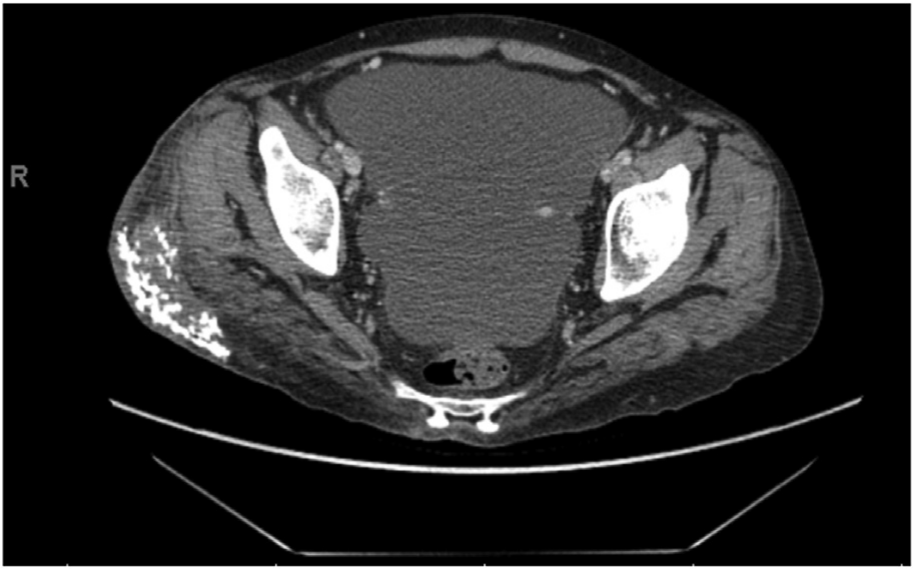
Computed tomography scan of patient’s pelvis obtained shortly after biopsy shown in Fig 1 with calcification in the *right* lateral thigh and gluteal subcutaneous fat. No underlying osseous destruction was noted.

On physical examination upon transfer, the right hip revealed an ulcer surrounded by a ring of dusky erythema and firm subcutaneous nodules (Fig 1, *A*). The patient appeared malnourished with loss of muscle mass and subcutaneous fat. Serum calcium and phosphorous levels were normal. A pelvic hip X-ray demonstrated a 6.4 by 7.4 cm lesion of packed calcification in the right hip with no erosion or destruction. A subsequent computed tomography of the affected area redemonstrated the foci of calcification (Fig 2).

**Fig 3:**
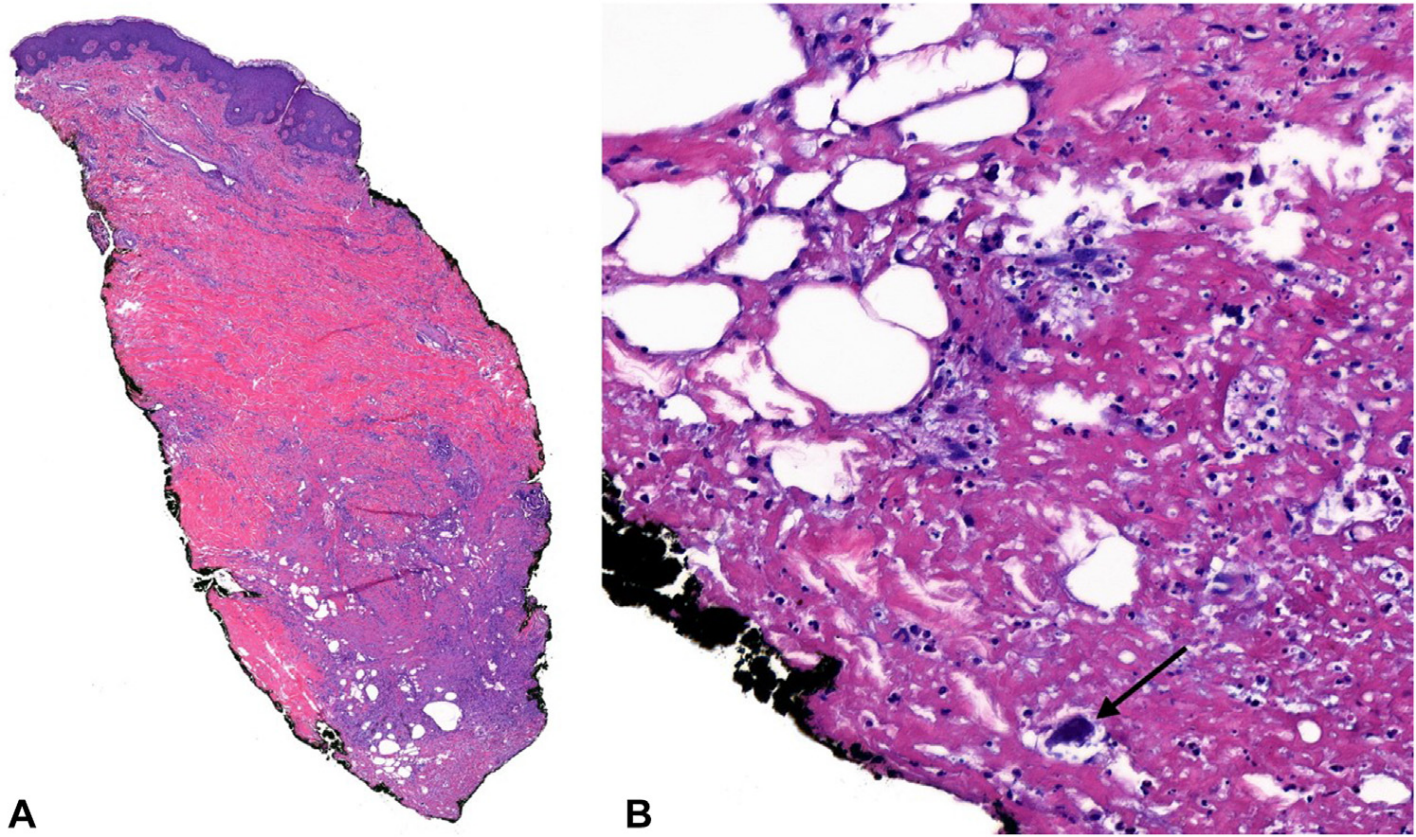
**A,** Histopathology scanning magnification of a shows necrosis of the subcutis with prominent fibrosis (Hematoxylin and eosin [H&E], 20× mag). **B,** At the base, lipomembranous fat necrosis with chronic degenerative changes is observed: foci of calcium deposit (*arrow*) and fibrin deposition. Calcification of the vessels was not observed (H&E, 400× mag).

Examination of outside pathology showed prominent subcutaneous calcification without intramural calcification or vasculopathy. During her hospitalization, the patient underwent two punch biopsies, each displaying suppurative granulomatous fibrosing dermatitis with focal tissue calcium deposits (Fig 3, *A* and *B*). Calcium deposits were not visualized within the vasculature on either biopsy or additional step sections (Fig 3, *B*). Collectively, the intermittently tender nodules, normal serum calcium levels, and lack of intramural calcification led to a final diagnosis of dystrophic calcification.

A multidisciplinary approach was used in addressing the patient’s comorbidities, including malnutrition and Wernicke’s encephalopathy. Given the initial concern for calciphylaxis, our patient was started on intravenous (IV) sodium thiosulfate (STS) 25 mg three times weekly. Due to severe nausea, the dosage was reduced to 12.5 mg and trimethobenzamide was administered prior to infusions with improvement. Intralesional STS was considered but not available. Surgical management was avoided due to the patient’s malnourished state and encephalopathy. The wound demonstrated gradual improvement during her hospitalization (Fig 1, *B*).

## Discussion

This case suggests that overlap in presentation of dystrophic calcification and calciphylaxis may create diagnostic challenges. Nonuremic calciphylaxis has been associated with alcoholic cirrhosis, rapid weight loss, and normal serum calcium levels, all of which were seen in our patient^1^^,^^4^, ^5^, ^6^ However, calciphylaxis is characteristically quite painful, while our patient’s lesions were intermittently tender^1^^,^^2^ Ultimately, the absence of vascular calcification in multiple biopsies and the presence of subcutaneous calcification led to the final diagnosis of dystrophic calcification over calciphylaxis.

Dystrophic calcification can occur in the setting of connective tissue (CT) disorders, panniculitis, neoplasms, infections, trauma, and burns that result in CT damage.^1^^,^^2^ Dystrophic calcification is most commonly seen in systemic sclerosis, dermatomyositis, and systemic lupus erythematosus.^7^ An autoimmune workup of our patient was negative for antinuclear antibodies (<1:40), anti-smooth muscle antibodies, anti-neutrophil cytoplasmic antibodies, perinuclear anti-neutrophil cytoplasmic antibodies, anti-Sjogren Syndrome A antibodies, anti-Sjogren Syndrome B antibodies, and mitochondrial antibodies. While our patient had no known risk factors for dystrophic calcification, given the patient’s encephalopathy, trauma of unknown origin cannot be ruled out as a potential contributing factor. With several risk factors for calciphylaxis present and an apparent lack thereof for dystrophic calcification, histopathology was critical to our final diagnosis. This case highlights the need for critical assessment of patient history, physical examination, laboratory and imaging results, and histology to arrive at a final diagnosis.

As the subtypes of calcinosis cutis can present on a clinical spectrum, circumstances may necessitate the consideration of STS as a therapeutic option. IV STS is a widely used treatment for calciphylaxis, a condition whose poor prognosis makes early treatment critical.^1^^,^^4^^,^^8^ Two reports of successful treatment of dystrophic calcification with IV STS exist: 1 in a 14-years-old with juvenile dermatomyositis and another in a 54-years-old with dermatomyositis and systemic lupus erythematosus.^7^^,^^9^ However, a retrospective case report of three patients with treatment resistant, CT disease-related dystrophic calcification found no clinical improvement with IV STS.^10^

Intralesional STS, which lacks some systemic side effects of IV STS, such as nausea, has also been employed in the treatment of dystrophic calcification, although research regarding its efficacy is limited. In a double-blind, placebo-controlled pilot study, only one of four patients with dystrophic calcification demonstrated a response to intralesional STS.^11^ A patient with refractory digital dystrophic calcification successfully treated with both IV and intralesional STS has also been reported.^12^ Additional research is needed to determine which patients may benefit from IV vs intralesional STS. Our case adds to growing evidence indicating IV STS may serve as a viable treatment for dystrophic calcification.

## Conflicts of interest

None disclosed.
